# Cross-platform Analysis of Cancer Biomarkers: A Bayesian Network Approach to Incorporating Mass Spectrometry and Microarray Data

**Published:** 2007-04-29

**Authors:** Xutao Deng, Huimin Geng, Hesham H. Ali

**Affiliations:** 1 College of Information Science and Technology, University of Nebraska at Omaha, Omaha, NE 68182, U.S.A; 2 Department of Pathology and Microbiology, University of Nebraska Medical Center, Omaha, NE 68198, U.S.A

## Abstract

Many studies showed inconsistent cancer biomarkers due to bioinformatics artifacts. In this paper we use multiple data sets from microarrays, mass spectrometry, protein sequences, and other biological knowledge in order to improve the reliability of cancer biomarkers. We present a novel Bayesian network (BN) model which integrates and cross-annotates multiple data sets related to prostate cancer. The main contribution of this study is that we provide a method that is designed to find cancer biomarkers whose presence is supported by multiple data sources and biological knowledge. Relevant biological knowledge is explicitly encoded into the model parameters, and the biomarker finding problem is formulated as a Bayesian inference problem. Besides diagnostic accuracy, we introduce reliability as another quality measurement of the biological relevance of biomarkers. Based on the proposed BN model, we develop an empirical scoring scheme and a simulation algorithm for inferring biomarkers. Fourteen genes/proteins including prostate specific antigen (PSA) are identified as reliable serum biomarkers which are insensitive to the model assumptions. The computational results show that our method is able to find biologically relevant biomarkers with highest reliability while maintaining competitive predictive power. In addition, by combining biological knowledge and data from multiple platforms, the number of putative biomarkers is greatly reduced to allow more-focused clinical studies.

## Introduction

Biomarkers, in the context of cancer diagnosis, usually refer to specific genes and their products which are indicators of disease states and can be detected in clinical settings. Microarrays and mass spectrometry, a pair of complementary tools for studying genome activity and proteome activity respectively, have emerged to bring hopes for discovering biomarkers and building diagnosis models. The idea is to screen genome or proteome activity with microarray or mass spectrometry to find a panel of biomarkers (usually five to 20) and use them to build a diagnosis model that could outperform established single-protein biomarkers, such as PSA (Prostate Specific Antigen) for prostate cancer and CA-125 (Cancer Antigen) for ovarian cancer (Diamandis, 2004).

The large-scale screening of genes and their products made the technologies extremely appealing not only for diagnosis but also for finding treatment for the diseases. Numerous studies have been performed on data sets using either microarray (Liu et al. 2005; Golub et al. 1999; Statnikov et al. 2005; Singh et al. 2002) or mass spectrometry (Lilien et al. 2003; Petricoin, 2002b; Petricoin et al. 2002a; Wagner et al. 2004; Liu and Li, 2005) technology. Many of these studies showed performance superior to current clinical biomarkers such as PSA for prostate cancer diagnosis. Although the biotechnology behind microarrays is fundamentally different from that of mass spectrometry, the strategies for biomarker finding and predictive model building are similar. They can be considered as a three-step data mining procedure. 1. Data generation and preprocessing: both healthy and ill patients’ data are collected; the data are usually preprocessed by normalization, outlier detection, baseline correction (in mass spectrometry), etc. 2. Computational biomarker extraction: standard tools such as ANOVA (ANalysis Of VAriance), t-test, PCA (Principal Component Analysis) and GA (Genetic Algorithm) can be used to select a small panel of genes in microarray or mass-to-charge ratios (*m*/*z*) in mass spectrometry. 3. Classification model building: standard classification tools, such as SVM (Support Vector Machine), DT (Decision Trees), DL (Decision List), kNN (k-Nearest Neighbors), etc., are routinely used to build predictive models based on selected biomarkers. Note that in some studies, steps 1 and 2 or steps 2 and 3 are combined.

Our study focuses on the biomarker extraction step because the quality of the extracted biomarkers holds the potential for reliable diagnosis and treatment of the diseases. The extraction of consistent and biologically relevant biomarkers can also significantly improve our understanding of the disease and its mechanisms. However, many issues exist in this area due to either technology limitations or computational artifacts which have been extensively discussed in the literature (Diamandis, 2004; Conrads et al. 2003; Sorace and Zhan, 2003). For example, several studies showed inconsistent sets of biomarkers extracted for prostate cancer (Diamandis, 2004; Sorace and Zhan, 2003; Baggerly et al. 2004). Lacking confirmation of disease-specific biomarkers posed a huge problem in the clinical application of both mass spectrometry and microarray data.

From the data mining point of view, biomarkers extraction from both mass spectrometry and microarray suffers from “curse of dimensionality” (Bellman, 1961); that is, the number of candidate biomarkers (usually thousands) is much greater than the number of samples or patients (usually dozens) in the study. A direct implication is that the search space for candidate biomarkers is too large for the small number of constraints so that the resultant biomarker solution will not be stable. An analogy is that, in a linear equation system when the number of unknown parameters is greater than the number of equations, there could be multiple (or an infinite number of) solutions. This inconsistency indicates that the biomarkers are largely determined by the search algorithm used in the study. It was also reported that the cryptic biomarkers or noise peaks could result in “good” classification of healthy and cancer samples (Sorace and Zhan, 2003).

The quality of selected biomarkers is usually determined by their predictive power—that is, accuracy in predicting new samples. Due to the foregoing reasons, we introduce another quality measurement of biomarkers, *reliability*, which is defined as the probability of a gene being a true biomarker given the experimental data. Biomarker extraction strategies include *filters*, which select biomarkers independent of the choice of classifiers, and *wrappers*, which search biomarkers by optimizing given classifiers. We take the filter approach because wrappers are classifier-dependent and tend to capitalize on chance especially for large-scale microarray and mass spectrometry data (data having dimension curse). Therefore, our goal is not to invent another search algorithm which picks up one set of biomarkers from possibly many biomarker sets. Instead, our approach is designed to narrow down the number of candidate biomarkers so that their presence is supported by multiple experimental platforms. While previous studies focus on extracting biomarkers from either microarray or mass spectrometry data sets independently, our approach is to associate microarray and mass spectrometry biomarkers to cross-validate their existence by the evidence from each. We use multiple data sets including mass spectrometry and microarray profiles to introduce more constraints to the biomarker extraction system. In addition, we apply available biological knowledge in the biomarker extraction process to further limit the search space and obtain a very small list of biomarkers.

We introduce the concept of *reliability*, which is the posterior probability of a protein being a biomarker after seeing the experimental mass spectrometry and microarray data. This Bayesian concept is mathematically defined in section 3 and calculated using our proposed Bayesian network (BN) model. Intuitively, if a gene biomarker shows evidence in microarray data and its associated peaks also appear in the mass spectrometry markers, this biomarker shows high reliability and we have greater belief that the gene and its products could be a reliable biomarker. In addition to the computational prediction accuracy, reliability is another performance measurement of putative biomarkers and it is very important in multi- or cross-platform biomarker analysis.

We find that the BN model and its algorithms are especially effective in integrating multiple heterogeneous data and performing biomarker extraction and analysis. Relevant biological knowledge can be built into the model by the use of proper prior parameters and appropriately designed network structure. The biomarker extraction problem can be nicely formulated as a probability inference problem and efficiently solved using an existing inference algorithm. To connect specific proteins and mass spectrometry data, an algorithm is developed by simulating post-translational modifications (PTMs) of proteins. The computational results show that our method can find biomarkers with the highest reliability while maintaining competitive predictive power. In addition, by combining biological knowledge and data from multiple platforms, the number of candidate biomarkers is greatly reduced, allowing more-focused on clinical studies.

## Study design and data processing

**Figure 1.**
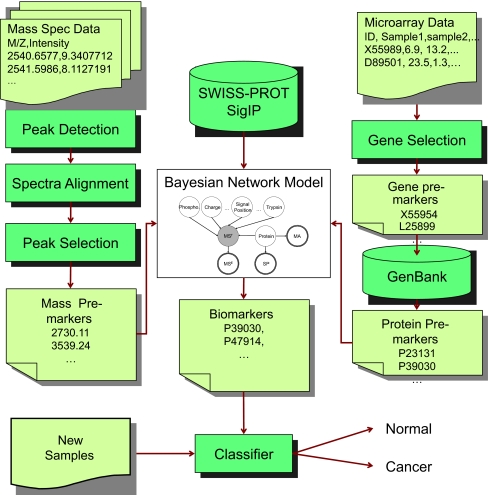
Study design of biomarkers extraction and their application in disease prognosis. Peak detection, alignment, and peak selection are performed on the mass spectrometry data. Gene selection is performed on microarray data. Then the pre-biomarkers are filtered using a Bayesian network model to obtain final biomarkers

The flowchart of this study is displayed in Figure 1. The raw mass spectrometry and microarray data are pre-processed through a few steps and become *pre-biomarkers*. Basically the pre-biomarkers are genes or peaks that are expressed differentially in cancer and healthy samples. The pre-biomarkers are used for inferring biomarkers in our BN model. The details of each component in the flowchart are explained in the following sections.

### Data description

We applied our method to the data sets containing both microarray and mass spectrometry data from prostate cancer patients and healthy controls. The microarray data was obtained from Singh et al. (2002) and the mass spectrometry data from Petricoin et al. (2002b). The microarray data set contains high-quality expression profiles obtained from 52 prostate tumor samples and 50 prostate non-tumor samples, using oligonucleotide microarrays containing probes for 12,600 human genes and ESTs (Expressed Sequence Tags). The mass spectrometry data set contains 69 cancer samples (26 samples with PSA level 4–10 ng/mL and 46 samples with PSA level greater than 10 ng/mL), and 63 normal samples with no evidence of cancer (PSA level less than 1 ng/mL). It was collected using an H4 protein chip and a Ciphergen PBS1 SELDI-TOF (Surface-Enhanced Laser Desorption and Ionization Time of Flight) mass spectrometer. The data description can be found in (Petricoin et al. 2002b) and the data can be downloaded at http://home.ccr.cancer.gov/ncifdaproteomics/ppatterns.asp. It is important to note that the mass spectrometry samples are from serum while the microarray samples are from prostate tissues. The spectra were exported with the baseline subtracted. The *m*/*z* values range from 0 to 20,000. The sample proteins were not processed by external proteases such as trypsin. However, serum proteins are frequently found to be cleaved by chymotrypsin, trypsin and elastase (Richter et al. 1999) so that the mass spectrometry data reflect cleaved protein segments rather than intact proteins.

Before we make use of the BN model to find reliable biomarkers, the microarray and mass spectrometry data were first independently cleaned, adjusted, and transformed into a form that is able to be processed by a BN. We performed peak detection and peak alignment on the raw mass spectrometry data, and extracted pre-biomarkers from both mass spectrometry and microarray data. Pre-biomarkers, as the final preprocessed data sets, refer to the differentially expressed genes or peaks in cancer and control samples.

### Peak detection from mass spectra

The raw spectrum for each sample is composed of 15,154 (*x*, *y*) pairs, where x axis records *m*/*z* values with corresponding intensity on *y* axis. Therefore, we have 15,154 features for only 132 samples. Obviously the number of features is too large to build a reliable diagnosis model. The peak detection is the first step in reducing the number of features. Peaks are basically the features which have local maximum intensities. Current peak detections are usually made by the software bundled with a spectrometer and hence the algorithms are hidden from users. The algorithm we use here is very simple and straightforward: we register all the *m*/*z* values if the corresponding peaks (local maximum intensity) exceed user-specified thresholds. We use both absolute threshold (intensity from baseline) and relative threshold (intensity from both the left and the right hill feet of the peak). Both thresholds are empirically set by a human annotator at 0.3.

### Peak alignment

We applied the time-warping algorithm (Wang and Isenhour, 1987) to aligning the peaks extracted from each sample. The time-warping algorithm employs dynamic programming and is very similar to the global sequence alignment algorithm. After peak detection and alignment, the mass spectra still contain 6,467 features (aligned peaks) with *m*/*z* value above 1,000. The number of features is further reduced to 5,709 by requiring that a peak must be observed in at least two samples to avoid noise peaks.

### Pre-biomarker (gene, mass peak) selection

Pre-biomarkers, as the final preprocessed data sets, refer to the genes or peaks that are informative in the sense that they are differently expressed in cancer and control patients. Selecting pre-biomarkers is a common practice which can significantly reduce the data dimension. The methods for extracting informative genes or mass peaks are essentially the same. In this study, we use the t-statistic with permutation test (Golub et al. 1999). For each gene or mass spectrum, we compute the t-statistic using two group labels. Then we randomly permute the labels 10,000 times to see whether the t-statistic is significantly correlated with class labels. The level of significance α for an individual test is set at 0.0005. Note that multiple statistical tests could result in many false-positive biomarkers by chance. To overcome this problem, we use Bonferroni correction to adjust the level of significance. This step yields 1,398 significant mass peaks (908 over-expressed and 490 under-expressed in cancer samples) and 240 gene (261 over-expressed and 175 under-expressed in cancer samples) as pre-biomarkers. Note that other selection criteria such as False Discovery Rate (FDR) (Benjamini and Hochberg, 1995; Pawitan et al. 2005), Fisher criterion, and F-statistics, can also be applied to biomarker feature selection.

## A BN Model for Biomarker Extraction

A BN (Pearl, 1998; Heckerman et al. 1995) is a graph-based model for representing probabilistic relationships between random variables. The random variables, which may represent data source such as gene expression levels, are modeled as graph nodes; probabilistic relationships are captured by directed edges between the nodes, and the conditional probability distributions associated with the nodes. The BN model is convenient for approximating complex joint distributions among several entities. A direct edge between two nodes indicates their direct dependency. Formally, a BN for a set of random variables is a pair *B* = <*G,* Ψ>, where the first component, *G*, is the network structure, and the second component, Ψ, is the numerical parameters for conditional distributions P(X|parents(X)) associated with each node X. A BN can be viewed as a probabilistic expert system which encodes a set of knowledge and makes inference based on observations.

### Biological rules

The preprocessed pre-biomarkers are the input (observations) to the BN model. The output would be the final cancer biomarkers of interest. The design and implementation of the BN model are guided by the biological knowledge extracted from literatures and from experts in cancer and blood biology. The rules are as follows:

1. *Over-expressed*: With a few exceptions, the most promising cancer biomarkers are over-expressed in cancer patients (Diamandis, 2004).
2. *Serum proteins*, which are the most promising biomarkers, contain a mixture of secreted proteins and species shed into blood circulation from diseased dying or dead cells present throughout the body (Conrads et al. 2003).
3. *Signal peptides:* Some proteins possess a *signal peptide*—a short peptide (15–60 amino acids long) that directs the post-translational transport of a protein. The existence of signal peptides is an important factor in determining whether a protein is secretory or not.
4. *Ionized peptides:* Ionized peptides generated in SELDI (Surface-Enhanced Laser Desorption and Ionization) mass spectrometry are predominantly singly charged (Siuzdak, 2003).
5. *Amino acid modification*: Certain amino acids can be modified by chemical groups. Common modifications include carboxylation, phosphorylation, oxidation, etc.
6. *Peptide fragments*: Peptide fragments, rather than intact proteins, are the predominant form (greater than 95%) in human serum (Adkins et al. 2002; Richter et al. 1999).
7. *Proteolytic cleavage*: Most proteins undergo proteolytic cleavage following translation. In particular, serum proteins are frequently found to be cleaved by chymotrypsin, trypsin and elastase (Richter et al. 1999).
8. *Signal peptide cleavage*: signal peptides must be cleaved to reflect the true mass spectra.

The first three rules are general and used to guide our overall BN model design. We are most interested in the genes with elevated mRNA levels detected by microarrays and those whose protein products are secreted into the circulatory system and detected by mass spectrometry as over-expressed peaks. To accommodate rule 1, we focus only on over-expressed genes and peaks in patients in this study. For rules 2 and 3, we explicitly model the influence of secretory proteins because they are a key factor in connecting the microarray and mass spectrometry data. We use the outputs from the SignalP 3.0 server (Bendtsen et al. 2004) to determine whether a protein is possibly secreted or not just based on its N-terminal sequence. The SignalP server uses an algorithm based on the Hidden Markov Model (HMM) (Nielsen and Krogh, 1998) which outputs the probability of a protein containing a signal peptide and the position of the cleavage site.

For rule 4, we simply assign a small probability to the peptides having double or higher charges. Rules 5–8 are mainly concerned about PTMs which significantly affect the mass spectrometry patterns. Many proteins are synthesized as inactive precursors that are activated under proper physiological conditions by limited proteolysis. A protein may undergo numerous PTMs so that it could be present in many forms at one time in the sample. Proteins and the mass spectrometry data are connected by theoretical mass spectra which are stochastically simulated using rules 4–8. The simulation is designed based on probabilistically modeling the biological knowledge about the modification of nascent proteins and the SELDI system. There is also some quantitative knowledge about PTMs. Specifically, about 30% of all mammalian proteins are phosphorylated (Liebler, 2001), and more than 90% of peptide fragments are proteolytically cleaved by trypsin, chymotrypsin or exoproteinase (Richter et al. 1999). These rules help us to assign proper prior probabilities for the PTM events.

### A BN model for biomarker finding

**Figure 2.**
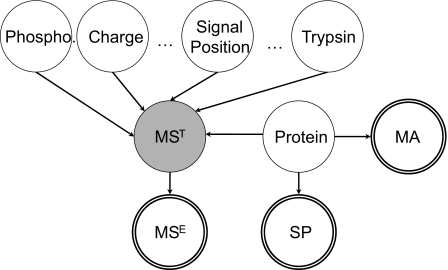
Proposed BN model for biomarker finding.

The data entities and knowledge are modeled as a BN shown in Figure 2. The variable “*Protein*” is to model the probability distribution of all proteins to be biomarkers. The prior distribution for *Protein* is uniform, indicating every protein has the same probability to be a biomarker. Since the choice of *Protein* determines its microarray pattern and its probability to contain a signal peptide, we have edges from the node *Protein* to nodes *MA* (microarray) and *SP* (signal peptide) respectively. The *Protein* node together with the *PTM* nodes determines the pattern of the theoretical mass spectra (denoted as *MS^T^*) of specific proteins. The variable *MS^E^* represents the experimental mass spectra which contain a mixture of a large number of proteins and peptides from serum. *MS^T^* is introduced to connect *Protein* with *MS^E^* and to help annotate the mass markers at the protein level. Single-circled nodes are hidden variables in the sense that their values are not observed by the experiments, while double-circled nodes indicate observed variables which are used to model the outputs from *MA*, *SP*, and *MS* (mass spectra) respectively. Node *MS^T^* is shaded to indicate it is a non-founder hidden variable. Nodes without parents are founder variables whose prior distributions need to be specified.

Beta distributions are used to model our prior belief of the occurrence of each PTM. (See supplementary information for Beta distribution and its simulation.) The prior distributions for the founder nodes are set up according to rules 4–8, and their effects on amino acids (AA) are displayed in Table 1. Each PTM event on the protein sequence can be treated as a Bernoulli trial with probability of success θ ~ Beta (α_1_, α_2_) where α_1_, α_2_ are parameters to specify the belief distribution. In order to use a BN for inference, we also need to specify the conditional probability distribution P(X|parents(X)) for every non-founder node X. We will discuss these conditional probability distributions in the next section.

The variable *MS^T^* is a hidden node. Its conditional distribution, given a protein sequence and all the PTM probabilities, is determined by simulation. *MS^T^* for a single protein is represented in the following format:

(1) $$MS^{T}=\{(mz_{j,}f_{j})|j=1,\ldots ,r\}$$

where *mz_j_* is the *m*/*z* ratio, *f_j_* is the frequency of the *j*th peak, and *r* is the number of peaks generated by simulation of the protein.

We use PRO to denote the set of all proteins in the universe of discourse. Other variables *MA*, *SP* and *MS^E^* are observed and they are used to model the outputs from the preprocessed experimental data sets. *MS^E^*, *MA* and *SP* are represented in the following format:

(2) $$\begin{array}{l} MS^{E}=\{mz_{i}|i=1,\ldots ,n\}\\ MA=\{t_{\text{protein}}|\text{protein}\in \text{PRO}\}\\ SP=\{p_{\text{protein}}|\text{protein}\in \text{PRO}\} \end{array}$$

where *n* is the number of pre-biomarkers of mass spectra after preprocessing; *t_protein_* is the *t*-score for the protein in the microarray experiments; and *p_protein_* is the probability of the protein containing a signal peptide generated by the SignalP 3.0 server.

Using our BN model, the biomarker extraction problem can be regarded as a classic Bayesian posterior probability inference problem. The posterior probability of interest is the probability of a protein given the observation of *MS^E^*, *MA*, and *SP*. The objective is to find a set of biomarkers with the highest posterior probabilities.

## Simulation and Scoring

The goal of the simulation algorithm is to generate *MS^T^* patterns by applying rules 4–8 as random events. For each amino acid in the protein sequence, we tested every PTM to see whether a PTM is possible at that position. If it is possible, we draw a sample θ from Beta (α_1_, α_2_) as the prior distribution for that PTM. Then we performed a Bernoulli trial with a probability of success θ. If the trial is successful, we perform the PTM on the protein sequence; otherwise we skip the PTM. We repeated the simulation 1,000 times and recorded the peak distribution as *MS^T^* for that protein.

**Simulation Algorithm**

**Input:** a protein sequence and the prior distribution of all PTMs where residue position is indexed by *i*, PTM is indexed by *j*.

**OutPut:** *MS^T^* for the protein

**for** (each position *i* in the protein sequence)

  **for** (each PTM *j*)

    **if** (position *i* meets the constraint of PTM *j*)

      1. Draw X ~ Beta (α_1_^,^ α_2_);

      2. Draw Y ~ U(0,1);

      3. if Y < X, perform modification; otherwise, no modification;

    **endif**

    **else** try next PTM *j* + 1;

  **endfor**

  go to next position *i* + 1;

**endfor**

**Figure 3.**
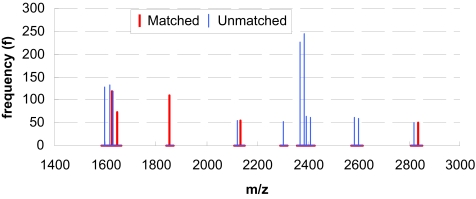
Example of *MS^T^* simulation results. The range of *m*/*z* values between 1,400 and 3,000 is magnified to show the detail of *MS^T^* pattern.

When the end of the protein sequence is reached, record *m/z* of simulated peptides in *MS^T^*;

Repeat the above procedure 1,000 times and output *MS^T^*;

Figure 3 shows an example *MS^T^* simulation of a protein with GI 9955963. Note that the *y* axis shows the frequency of each peak in 1,000 simulations (not the intensity of standard mass spectra outputs). A red peak (bold line) means the peak matched a peak in the *MS^E^* pre-biomarker, while a blue one (thin line) means unmatched. By simulating and matching *MS^T^* against *MS^E^*, we built the connection between a protein and experimental mass spectra.

The BN allows us not only to conveniently represent the biological knowledge but also to analyze and formalize the problem of biomarker extraction. The posterior probability of a protein, which is of our interest, is computed as follows

(3) $$\begin{array}{l} P(\text{protein}|MS^{E},MA,SP)\\ =\alpha \;P(\text{protein},MS^{E},MA,SP)\\ =\alpha \;P(MS^{E},MA,SP|\text{protein})P(\text{protein})\\ =\alpha^{\prime }\;P(MA|\text{protein})P(SP|\text{protein})\\ \;P(MS^{E}|\text{protein}) \end{array}$$

where α and α′ are constants independent of the choice of proteins. The first and second equations directly follow Bayes’ theorem while the third equation follows the conditional independence defined by our BN model structure. The last factor can be simplified as follows:

(4) $$\begin{array}{l} P(MS^{E}|\text{protein})\\ =\sum_{MS^{T}}P(MS^{E},MS^{T}|\text{protein})\\ =\sum_{MS^{T}}P(MS^{E}|MS^{T},\text{protein})\\ \;P(MS^{T}|\text{protein})\\ =P(MS^{E}|MS^{T}) \end{array}$$

where the first and second equations follow the definition of marginal distribution; the third equation is validated by observing that *MS^T^* is a deterministic variable given the protein sequence so that *P*(*MS^T^|_protein_*) =1; and *MS^E^* is independent of the protein given *MS^T^*.

The above analysis indicates that the score of a protein can be decomposed into three parts: its likelihood containing a signal peptide, its microarray profile, and its mass spectra pattern. Since the likelihood terms are difficult to define and estimate (except for *SP*), we further generalize this equation into a form that can be determined empirically,

(5) $$\begin{array}{l} S(\text{protein})=S(MA|\text{protein})S(SP|\text{protein})\\ \;\;\;\;\;\;\;\;\;\;\;S(MS^{E}|\text{protein}). \end{array}$$

This score *S*(*protein*) is called the *reliability* of biomarkers which is a generalization of the posterior probability in Equation (3). Now the problem of biomarker finding can be formalized to finding the set of proteins which have the highest reliabilities:

(6) {protein|S(protein)>σ}

where σ is a user-defined threshold to determine whether a reliability of a protein is high enough to make this protein a candidate biomarker.

The score for *SP* is defined as follows:

(7) $$S(SP|\text{protein})=p_{\text{protein}}$$

where *p_protein_* is the output of the HMM model in the SignalP Server.

Since we are only interested in over-expressed genes, we use a sigmoid function to define the score for *MA* outputs. See Figure S1 in supplementary information for an illustration of the function:

(8) $$S(MA|\text{protein})=\frac{1}{1+\text{exp}(-t_{\text{protein}}+\sigma )}.$$

To define the likelihood term *S*(*MS^E^|_Protein_*), we first define *S'*(*MS^E^|_Protein_*), which is the raw score of observing the experimental spectra given a protein,

(9) $$\begin{array}{l} S^{\prime }(MS^{E}|\text{protein})=S^{\prime }(MS^{E}|MS^{T})\\ =\frac{\text{WSPC}}{\text{length}}=\frac{\sum_{f_{j}}\left(f_{j}|f_{j}>\delta \;\text{and}\;mz_{j}\in MS^{E}\cap \;MS^{T}\right)}{\text{length}} \end{array}$$

where *length* is the number of amino acids contained in the protein; *MS^E^* ∩ *MS^T^* denotes the set of peaks shared (within a certain threshold) by the two spectra. *WSPC* (Weighted Shared Peaks Count) is the sum of all frequencies of the shared peaks greater than a certain threshold δ. *WSPC* is then divided by the *length* of the protein to eliminate the dependency between the raw score and *length* (see Figure S2 in the supplementary information).

The score S(MS^E^|protein) is further normalized by dividing its raw score by the supremum of the scores for all proteins.

(10) $$S(MS^{E}|\text{protein})=\frac{S^{\prime }(MS^{E}|\text{protein})}{{\text{sup}}_{{\text{protein}}^{\prime }\in \text{PRO}}S^{\prime }(MS^{E}|{\text{protein}}^{\prime })}$$

We see that all the scoring definitions are properly normalized into the interval [0, 1]. Although some of them may not be interpreted to be probability, they could be used as empirical measurements for the extent of matching between a protein sequence and experimental results.

## Biomarker Analysis and Results

We implemented the pre-processing modules, the BN model and its associated algorithms in Microsoft™ Visual™ C++ 6.0. The post-processing and graphics was performed using Matlab™.

### Distributions and *p*-value

**Figure 4.**
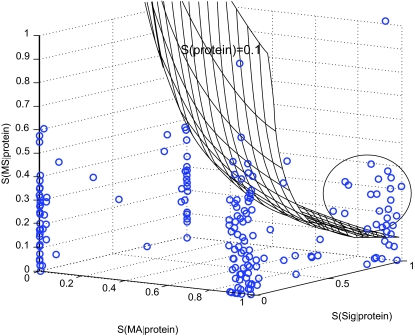
Pre-biomarkers distributed in a space defined by the scores on each data set (*MA*, *SP*, *MS*). Every point on the mesh has a score *S*(*protein*) = 0.1; above the mesh, *S*(*protein*) > 0.1; under the mesh, *S*(*protein*) < 0.1.

We applied the BN inference algorithm and the scoring scheme to 240 protein pre-biomarkers obtained from microarrays (See supplementary Table S1 for the list of pre-biomarkers). Figure 4 shows the scores plotted in the three-dimensional space with each dimension corresponding to one of the three components in Equation (5).

The 240 pre-biomarkers are roughly clustered at four of the corners in the space. There are only 32 proteins above the mesh, indicating their reliabilities (scores) higher than 0.1. We will discuss why 0.1 was used as a score standard to select candidate biomarkers in the following paragraphs. This group of 32 proteins is notated as candidate biomarkers and most of them are clustered into a sphere as indicated in Figure 4.

**Figure 5.**
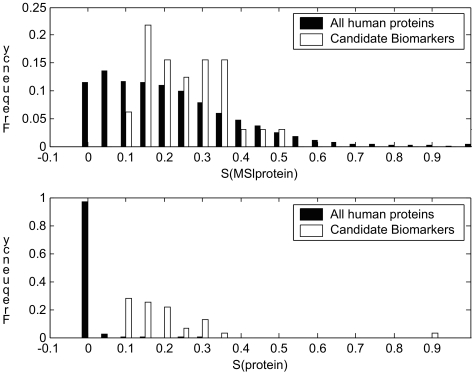
Distributions of candidate biomarkers and all human proteins. **a**. Distribution of *S*(*MS*|*protein*); **b**. Distribution of *S*(*protein*).

The BN method is also applied to all human protein sequences, including 12,484 sequences obtained from SWISS-PROT release 47.2 (Bairoch et al. 2005). The histograms in Figure 5 show the scoring distributions of all human proteins vs. candidate biomarkers. Figure 5a shows the scores for mass spectra *S*(*MS|protein*). We see that the scores for the population (all human sequences) are distributed in a shape similar to exponential distribution (*cases* = 12,484, *mean* = 2.59, *SD* = 2.22). The scores for 32 candidate biomarkers have a higher mean but the two distributions are not sufficiently separated (*cases* = 32, *mean* = 3.35, *SD* = 2.00). Figure 5b shows the *S*(*protein*) distributions of the candidate biomarkers and all human proteins. The candidate biomarker distribution is clearly separated from the population distribution and the number of candidate biomarkers is considerably reduced as a result of introducing multiple data sets in our BN model.

The distributions in Figure 5b could be used to construct a statistical test for the reliability. The null hypothesis (H_0_) states that a protein with a reliability score is randomly picked from the population, while the alternative hypothesis (H_1_) states that the protein is NOT randomly selected from the population. If we use the reliability as the test statistic, we observed from Figure 5b that a reliability score greater than 0.1 corresponds to a *p*-value less than 0.01. This is the reason we use 0.1 as a score standard to select candidate biomarkers. Caution should be taken when interpreting *p*-values. The statistical test determines whether a protein with a specific reliability is likely due to chance (H_0_) or significantly unlikely due to chance (H_1_). This indirect test is intended to simulate a direct test that determines whether the protein is a biomarker or not, but the two tests are not equivalent and the latter one can only be justified by experimental study. Figure 5 also shows that the separating powers of each data source are not equal. Especially, the mass spectrometry data are not as discriminating as microarray data and signal peptide data. This can also been seen in Figure 4, where microarray and signal peptide data clearly separate all the points into four groups.

### Sensitivity analysis

Although we specified the prior distributions according to biological rules, the BN model should be robust regardless of the choice of subjective parameters. Robustness is a characteristic of the model and its assumptions, while reliability is a characteristic of a protein given the model.

**Figure 6.**
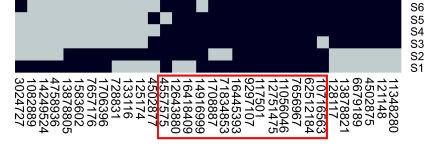
Sensitivity analysis results. Each column represents a biomarker and each row represents a parameter set. A black or gray square corresponds to presence or absence of a biomarker under a certain set of parameter.

The robustness was studied by sensitivity analysis of how the prior parameters’ changes influence the inference of biomarkers. We assigned six sets of values (S1, S2, …, S6) to the parameters (amino acid PTMs, cleavage PTMs, the threshold δ, and the number of simulation repeats). For each setting of parameters, we performed BN inference and selected 20 candidate biomarkers with the highest scores. The values of the six sets of parameters are displayed in Table 2 and the results of corresponding biomarkers are summarized in Figure 6. Not surprisingly, we found that the inference is relatively sensitive to the parameters of cleavage PTMs, as we observed that the biomarkers obtained with the parameter sets S1 and S2 diverge from those obtained with the other four parameter settings. However, 14 proteins are consistently found in at least five of the six sets, and six of them appear in all six parameters sets. These 14 biomarkers and their scores are summarized in Table 3.

All of the 14 proteins are over-expressed in cancer tissues and very likely to contain a signal peptide. All 14 proteins have significant reliability scores (*p*-values < 0.01) and relatively high mass spectrometry scores, indicating that their *MS^T^* (theoretical mass spectra) match *MS^E^* (experimental mass spectra) to a relatively large extent. An immediate finding is that PSA (APS) (in Table 3), probably the only biomarker that is employed in current clinical practice, is identified by our method. The identification of PSA as a reliable and robust biomarker strongly suggests that our method is effective in finding biologically meaningful biomarkers. We performed a manual OMIM (Online Mendelian Inheritance in Man) (OMIM, 2000) search for the functions of the 14 proteins. CANX and CRTC have a high affinity to binding calcium ions, indicating their roles in the regulation of signal transduction. Immunoglobulin IGSF4B and LU are cell adhesion molecules, and they play roles in cancer regulation (Fukuhara et al. 2001; Rahuel et al. 1996). CDH12 are calcium-dependent cell-cell adhesion molecules that may be involved in the metastasis and invasion of cancer. By matching *MS^T^* to *MS^E^* for the 14 proteins, we identified 20 unique peaks in the sense that each peak is associated with only one protein among the 14 proteins. The *m*/*z* values and their associated peptides are displayed in supplementary Table S2. A complete discussion of the 14 proteins is beyond the scope of this paper, but, they deserve further studies including biological experiments.

To test the classification accuracy, we performed a classification study on the set of biomarkers using SVM*^lignt^* (Joachims, 1999) as the classifier. The SVM*^lignt^* was set at default with a linear kernel. The leave-one-out estimates of the 14 protein biomarkers, 20 associated unique peaks, and PSA microarray data are displayed in Table 4. The classification accuracy shows that using panels of either microarray or their associated mass spectrometry biomarkers certainly outperforms using PSA as the sole biomarker. Our classification performance is comparable to the original reports (Petricoin et al. 2002b; Singh et al. 2002). They use searching-based strategies (wrappers) and search for biomarkers that maximize certain classifiers. However, our approach, instead of using a wrapper, uses a sophisticated filter which is classifier independent, and therefore less likely to be cryptic. In addition, our solution is supported by multiple biological evidences. Recall that, due to the “dimension curse”, there are many sets of biomarkers which have essentially equivalent discriminative power, and the determination of a solution depends on the searching path of a specific algorithm. But our approach could find the most reliable biomarkers at the meanwhile maintaining their diagnostic accuracies.

## Discussion

Our method built relationships between the biomarkers at both mRNA and protein levels, helping cross-validate the biomarkers obtained from different data sources. The candidate proteins revealed from our method, including PSA, are related to prostate cancer, which indicates our method is effective in finding biologically relevant biomarkers. By using data from multiple platforms, our method narrowed down thousands of putative gene biomarkers to the extent that the biomarkers can be studied individually. Among many thousands of genes, the list of gene biomarkers not only shows competitive predictive power but more importantly, shows the highest reliability meaning that their existence is supported by multiple sources of data.

Unlike the samples for peptide mass fingerprinting which are enzymatically digested proteins after being purified, our mass spectrometry samples are one-level-more complex because they consist of multiple unknown proteins which are then post-translationally modified *in vivo*. It is practically impossible to identify specific proteins from spectra made up of complexly mixed peptides as in serum. However, external controls (cancer vs. health samples), microarrays, and biological rules can facilitate the analysis and annotation of mass biomarkers. The greatest difficulty in using mass spectrometry data in our model is to associate individual proteins with the experimental mass spectrometry profile. We know that individual mass spectrometry peaks from mixture samples such as serum proteins cannot be accurately annotated to the sequence level without using tandem mass spectrometry. Our solution is to associate each individual protein NOT with individual peaks, but with the entire *MS* pattern so that the association of mass spectrometry data with each protein is not easily affected by individual peaks. This is done by simulating *MS^T^*, which reflects the existence of proteases and other PTM factors in serum, and comparing *MS^T^* with *MS^E^*. This strategy of linking proteins to mass spectra is supported by the fact that the protein biomarker PSA yields a large score (0.34) for its *MS^T^* compared with *MS^E^*. Admittedly, the existence and the use of biomarkers are not valid until they are clinically validated. However, with the resolution and sensitivity improvement of mass spectrometry technology, and with more cross-platforms, cross-labs becoming available, our computational model could shed more light into clinical studies.

Currently, there are no reliable computational methods to discriminate secreted proteins from membrane proteins. Some membrane proteins such as CANX (containing signal peptides) are inevitably selected by our method, although our goal is to find serum biomarkers. This example shows the importance of incorporating further biological knowledge into bioinformatics tools.

Distinguished with the methods focusing on applying search algorithms to a single data set, our method emphasizes the use of multiple data sets and biological knowledge to reduce the effect of bioinformatics artifacts and to enhance the reliability and relevance of biomarker searching. The merit of the BN model lies in its power to explicitly represent and compute biological knowledge and multiple entities.

Coombes et al. (2004) present a simulation-based approach to understanding the physical factors of mass spectrometry instruments and their effects on the mass spectra. Note that our proposed simulation algorithm mainly focuses on how the biological factors (such as trypsin and other PTMs) in human blood affect the mass spectrometry patterns of specific proteins.

One limitation of our current study is that we used only one data set from each type of experiments. It is possible that one error in the assay could change the conclusion on one biomarker. The power of our BN model lies in its ability to make inference on multiple observations—the more data it sees, the more accurate the results will be. To fully utilize the convenience of Bayesian models in biomarker analysis, we will include replicate data sets from multiple labs in future study.

The biomarker finding and its clinical use is a very complex issue, and a completed discussion is beyond the scope of this article. Refer to Lyons-Weiler (2005) for a review of the research in biomarker informatics. According to Pepe et al. (2001), our study is at the data exploratory phase of biomarker development and validation. Clinical assays and follow-up analysis are needed to confirm our finding of prostate cancer markers.

In this study, we focused on integrating microarray and mass spectrometry data and made inference of biomarkers using a BN model. However, our approach is not limited to such usages. With the fact that biomarkers are now generated from different types of instruments, multiple labs, various sets of samples and controls, the data analysis of biomarkers will be becoming increasingly complex and the integration and meta-analysis of multiple results will be attracting more attention in the near future. Our proposal of Bayesian strategy and the concept of reliability are important in evaluating biomarkers generated from heterogeneous platforms.

In the proposed model, unknown patient samples are scored according to a scoring system that is derived from gene-expression patterns of all training samples. Biomarkers based on a subset of samples or individual samples could allow the detection of subtle difference between subsets of samples and increase the overall prediction accuracy (Lyons-Weiler et al. 2004). The cross-validation results, shown in Table 4, may be optimistic, because testing data and training data come from the same experiments so that the variances caused by instrumental and experimental factors are not included in the analysis. The use of independent test data sets will be another important step to further validate the prediction performance of cancer biomarkers (Lyons-Weiler, 2005).

We must also point out that the current biological knowledge base in the BN model is a limited version; we included a few general biological rules and three numerical data sets. We are currently working on expanding and improving the current BN model to incorporate gene-specific information such as PubMed, OMIM, and GO. It has been shown that BN models are flexible and expandable to deal with complicated biological data types and data structures (Deng et al. 2005). Our long-term goal is to build a more intelligent knowledge engineering system which can “understand” complicated knowledge and extract biomarkers for more diseases.

## Supplementary Information

Figure S1. Sigmoid function to define the S(MA|protein) from t-value.

Figure S2. The effect of protein length on the scoring of MS. Coefficient of determination R2 drops from 0.72 to less than 0.01 by normalizing the score by the length. Clearly the linear dependency is largely eliminated by the normalization.

Beta Distribution and its Simulation Beta distribution is denoted by Beta (α1, α2) where α1, α2 are parameters to specify the belief distribution. Beta (α1, α2) has the mean $\frac{\alpha_{1}}{\alpha 1+\alpha 2}$ and the variance $\frac{\alpha 1\cdot \alpha_{2}}{{\text{(}\alpha 1+\alpha 2)}^{2}\text{(}\alpha 1+\alpha 2+1)}$. For example, Beta (2, 3) express that the probability (belief) of the occurrence of the PTM is centered at $\frac{2}{3+2}=0.4$, and the standard deviation of this belief is $\sqrt{\frac{2\cdot 3}{{\left(2+3\right)}^{2}(2+3+1)}}=0.^{\prime }.$; Beta (20, 30) also has mean $\frac{20}{30+20}=0.4$, but has a much smaller standard deviation $\sqrt{\frac{20\cdot 30}{{\left(20+30\right)}^{2}(20+30+1)}}=0.069$. Therefore, Beta distribution cannot only describe our belief of the occurrence of an event but the degree of uncertainty of our belief as well. The simulation of Beta distribution is performed via simulating Gamma distribution (See C++ code below).

**Table:** 

double Beta (double alpha1, double alpha2)	double Gamma (double alpha)
{	//gamma(alpha, 1), alpha >1
double y1 = Gamma(alpha1);	{
double y2 = Gamma(alpha2);	double a = 1/sqrt(2*alpha-1);
return y1/(y1 + y2);	double b = alpha-log(4.0);
}	double q = alpha+1/a;
	double theta = 4.5;
	double d = 1 + log(theta);
	B: double U1 = Uniform();
	double U2 = Uniform();
	double V = a*log(U1/(1-U1));
	double Y = alpha*exp(V);
	double Z = U1*U1*U2;
	double W = b+q*V-Y;
	if(W + d-theta*Z> = 0)
	return Y;
	else if(W> = log(Z))
	return Y;
	else
	goto B;
	}

**Table S1.** 240 pre-biomarkers obtained from MA, their t-score, and their HMM output.

**GI**	**t_protein**	**p_protein**
13904866	4.49	0.016
113950	−6.97	0.001
15431295	5.63	0
15055539	7.32	0
133041	6.52	0
4506607	4.75	0
1096944	4.75	0.001
136479	4.35	0
1082889	4.35	1
4557647	4.33	0
16753225	5.45	0.006
4506699	4.46	0
1096940	4.46	0
13904870	4.87	0
15220431	4.75	0
11415026	6.34	0
14591909	4.55	0
266921	5.69	0
1350706	5.77	0
4506713	4.20	0
133825	4.92	0
15718687	4.67	0
16579885	4.51	0.001
2493600	−4.10	0.002
116533	−5.06	1
1095781	−5.06	0.986
1708274	5.47	0
7512733	5.47	0.01
9910418	−4.45	0
128117	4.71	0.998
4557575	5.18	0.97
12229574	−6.61	0.998
25402878	−6.61	0
7108362	5.72	0
4759146	−4.65	0.996
4557543	−4.05	0.998
5803003	−4.06	0.002
17366160	4.73	0.064
17433099	−4.21	0
7657603	5.61	0
9955963	9.00	0.024
7656967	4.10	1
17374817	−4.23	0.045
121148	4.72	0.726
4885645	−5.26	0
113954	4.11	0
2497437	4.15	0.351
12585545	4.47	0.001
11352059	4.47	0
12643308	5.28	0
1345650	−4.99	1
4826768	4.69	0
4506701	−4.13	0
17978471	4.23	0
10190714	−4.68	0.003
14602449	−4.45	1
7512879	−4.45	0.045
11321634	4.48	0
5921743	−4.19	0.005
6005824	−4.58	0.024
11321603	−5.52	0
4507547	4.79	0.001
7662254	−4.58	0.001
7512876	−4.58	0.986
18490978	−5.60	0.367
11356305	−5.60	0.115
11360104	−5.60	0.001
9297107	4.06	0.999
50403775	−4.37	0.129
3914303	−6.61	0.043
2495731	−4.83	0
18699734	4.19	0
115945	−4.36	1
13626119	−4.82	1
123057	10.40	0.278
399193	−4.01	0
7657176	4.97	1
4758626	−4.03	0.004
12230067	−4.96	0.005
133486	−5.75	0.001
132387	−6.07	0
13129026	4.04	0
4503537	4.64	0
68068024	5.93	0
13129064	−5.13	0.006
1585496	−5.13	1
8134636	−5.25	0
121735	−4.96	0.015
4502109	−4.72	0
4885559	−4.43	0.029
11181775	−4.10	0
5453722	4.20	0.373
14165437	4.12	0
11993943	−4.19	0
13878821	4.29	0.75
2135080	4.29	0
1082633	4.29	0
728834	−4.88	0.962
10720334	5.12	0
127442	5.75	0.235
1708887	4.97	0.999
10716563	5.79	0.999
1351211	4.32	0
113463	6.45	0.049
14249524	4.79	0.999
16418409	4.52	0.998
10445223	4.63	0
6679189	5.17	1
133948	5.27	0.014
117098	5.16	0.449
125174	4.53	0.996
5902014	4.97	0.005
4504631	−4.18	0.002
1173039	4.32	0
1172922	4.27	0
4505581	−4.33	0
728833	−4.67	0.962
4506699	4.46	0
118504	−4.56	0.845
56405387	−5.21	0
135304	4.70	0
133701	−4.72	0.004
4505623	−4.87	0.008
11291391	−4.87	0
11360002	−4.87	0.003
127983	5.35	0
7661670	5.50	0.001
4502875	6.55	0.805
13432136	4.97	0.353
114322	6.07	0.001
13878805	4.04	0.997
10863909	4.03	0.999
25398579	4.03	0
5902020	−5.38	0
4506427	−5.37	0.999
12643622	7.07	0.627
7512940	7.07	0.004
728831	7.81	0.902
4758792	4.18	0.215
10835025	5.25	0.072
4502877	4.21	0.978
6912682	−4.88	0.999
71834857	4.98	0.998
13638228	4.93	0.004
9994169	4.66	0.001
18104976	4.07	0.703
8393299	−4.23	1
5453603	4.91	0.092
14916573	4.81	0
5031597	4.39	0.001
4758950	4.60	0.863
11056046	4.97	1
13637934	−4.94	0
25387602	−4.94	0
1703205	4.04	0
417246	5.19	0.002
119172	6.46	0
4507877	−5.34	0
14916999	4.16	1
1706396	4.46	0.884
5803145	4.35	0
1345695	−4.11	0
231741	−5.46	0
4758032	4.52	0
4507357	−4.04	0
6166568	4.21	0
13994151	−4.42	0
14548187	−5.17	0
4885509	−4.34	0.997
129483	4.32	1
7657552	5.37	0.007
1583602	5.37	1
1589585	5.37	0.157
61252057	−5.02	0.99
16306550	−5.58	0
5174485	−4.47	0.999
4557617	−4.62	1
114392	−4.35	0
16445393	10.05	0.999
133116	4.19	0.581
231475	−4.62	0
6005924	−7.54	0
115601	−5.92	0
2495724	−4.68	0
4505835	−5.17	0.541
17380550	−4.16	0.006
4757902	−5.67	0
14149680	−4.04	0
3024727	5.20	0.999
50403771	4.54	0.051
399866	4.88	0
13124879	−4.47	0
6226951	4.14	0
5453541	5.68	0.999
4826878	−4.21	0
5729836	4.63	0
1705731	−5.07	0.031
7513030	−5.07	0.001
8923881	4.34	0
3915626	−7.40	1
9257222	−4.77	0
1705650	4.92	0
12643880	4.09	0.958
10047134	−5.06	0
68846235	5.49	0
121110	−4.54	0.995
8923444	4.08	0
126047	5.81	0
5453736	−6.37	0
62512184	4.95	1
2135919	4.95	0
117501	4.60	1
5032159	−4.59	0.042
55977848	4.51	0.557
11348280	4.51	0.895
13878450	−5.14	0
4505037	−6.97	0.868
6912268	−6.26	0.02
131762	4.74	0
127983	5.35	0
226527	5.35	0
4557355	−4.41	0
4758936	4.70	0.996
17402909	−4.46	0
12751475	4.82	0.992
1730015	5.55	0.427
11352548	5.55	0
4557617	−4.62	1
226527	−4.62	0
223828	−4.62	0.722
17380263	4.12	0.014
2842764	4.02	0.989
113950	−6.97	0.001
10835023	−4.30	0.007
1352464	−4.16	0.001
4758594	−4.31	0.992
4557413	−4.46	0
125969	5.58	0
118295	4.50	0
2829468	−4.69	0

**Table S2.** Specification of 14 biomarkers identified by our BN mothods.

**GI**	**Description**	**Unique mass marker with peptide sequence**	**m/z (Da)**
16418409	pro-oncosis receptor inducing membrane injury gene ( **PORIMIN**)	TTSVSQNTSQJSTSTM(4)TVTHNSSVTTAASSVTI TTTMHSEAKK	4347
12643880	STK39_HUMAN STE20/SPS1-related proline-alanine rich protein kinase ( **STK39**)	VKEENPEIAVSASTIPEQIQSLSVHDSQGPPNANE DYR	4148

		APAPAAPAAPAPAPAPAPAAQAVGWPIC(1)RDAYE	4802
		LQEVIGSGATAVVQAAL	
71834853	prostate specific antigen isoform 3 preproprotein ( **PSA**)	QCVDLHVISNDVC(1)AQVHPQK	2291
16445393	cadherin 12, type 2 preproprotein ( **CDH12**)		–
7656967	cadherin EGF LAG seven-pass G-type receptor 1 ( **CELSR1**)	PVVHIQAVDADSGENARL	1891
		SFAGPIGAVIIINTVTSVLSAKVSCQRK	2859
		YVV(5)GWGIPAIVTGLAVGLDPQGYGNPDF	2897
		ADIGGMLPGLTVRSVVVGGASEDKVSVRRGF	3129
		DLAATQDADFHEDVIHSGSALLAPATRAAW	3149
11056046	immunoglobulin superfamily, member 4B ( **IGSF4B**)	GTYLTHEAKGSDDAPDADTAIINAEGGQSGGDD KKEYFI	4056
1708887	LU_HUMAN Lutheran blood group glycoprotein precursor ( **LU**)		-
9297107	NRP1_HUMAN Neuropilin-1 precursor (Vascular endothelial cell growth factor 165 receptor) ( **NRP1**)	GGIAVDDISINNHISQEDC(1)AKPADLDK	2896
		EGEIGKGNLGGIAVDDISINNHISQEDCAK	3096
		GM(4)ESGEIHSDQITASSQYSTNWSAERSRL	3243
62512184	SEL1L_HUMAN Sel-1 homolog ( **SEL1L**)	KPALTAIEGTAHGEPC(1)HFPF	2181
4557575	fatty acid amide hydrolase ( **FAAH**)		-
12751475	solute carrier family 39 (zinc transporter), member 6 ( **SLC39A6**)	DSQQPAVLEEEEVMIAHAHPQEVYNEY	3155
		GQSDDLIHHHHDYHHILHHHHHQNHHPHSHSQR YSREEL	4882
10716563	calnexin precursor ( **CANX**)	GTAIVEBHDGHDDDVIDIEDDLDDVIEEVEDSKPD TTAPPSSPK	4630
14916999	GRP78_HUMAN 78 kDa glucose-regulated protein precursor ( **GRP78**)	DVSLLTIDNGVFEVVATNGDTHLGGEDF	2934
		NTVVPTKKSQIFSTASDNQPTVTIKVYEGERPLT KDNHL	4356
117501	CRTC_HUMAN Calreticulin precursor (CRP55) (Calregulin) (HACBP) (ERp60) ( **CRTC**)	YTLIVRPDNTYEVKIDNSQVESGSL	2840

## Figures and Tables

**Table 1.** Founder nodes (Protein and PTMs), their effects on the mass spectra pattern and their prior distributions.

**Founder node**	**Affected AA**	*m/z***difference**	**Prior probability**
Carboxylation	C	+58.005479	Beta (2, 5)
Phosphory	T,S,Y	+79.966330	Beta (2, 2)
Water loss	S,T	–18.010565	Beta (2, 5)
Oxidation	M	+15.994915	Beta (2, 5)
Doubly charged	parent peptide	$\frac{\text{patent}\;\text{m}/\text{z}+1}{2}$	Beta (2, 10)
Trypsin	after K/R except before P	–	Beta (8, 2)
Chymotrypsin	after FLWY, except before P	–	Beta (8, 2)
Loss AA after cleavage	N-terminal, 1–4 AA lost	–	Beta (2, 10)
Signal Position	Determined by SignalP 3.0	–	Determined by SignalP 3.0
Protein	–	–	Uniform

**Table 2.** Six sets of parameter settings where S4 is the default setting.

**S3** **S6**	**S1** **S4 (Default)**	**S2** **S5**
**AA PTM Beta(**α**_1_,** α**_2_)**	(2,2)	(2,2)
(2,2)	(2,2)	(1,1)
(2,2)		
**Cleavage PTM Beta(**α**_1_,** α**_2_)**	(3,7)	(3,7)
(8,2)	(8,2)	(8,2)
(8,2)		
**Threshold** δ	20	50
20	50	20
20		
**Repeats**	1000	1000
1000	1000	1000
500		

**Table 3.** Biomarkers and their scores under default parameter setting.

**GI**	**Gene Name**	**Function**	**MS**	**MA**	**SP**	**Score**
16418409	PORIMIN	cell death	1.00	0.92	0.99	0.93
12643880	STK39	S/T kinase	0.40	0.88	0.95	0.34
71834853	PSA	Androgens regulated	0.34	0.95	0.99	0.32
16445393	CDH12	cell-cell adhesion	0.31	0.99	0.99	0.31
7656967	CELSR1	protein-protein interactions	0.33	0.89	1.00	0.30
11056046	IGSF4B(TSLC1)	Immuno-globulin	0.30	0.95	1.00	0.29
1708887	LU	Immuno-globulin	0.30	0.95	0.99	0.28
9297107	NRP1	cell growth factor	0.28	0.88	0.99	0.24
62512184	SEL1L	gene regulation	0.25	0.95	1.00	0.24
4557575	FAAH	fatty acid amides	0.21	0.96	0.97	0.20
12751475	SLC39A6	zinc transporter	0.21	0.94	0.99	0.19
10716563	CANX	calcium ion binding	0.18	0.97	0.99	0.18
14916999	GRP78	Protein folding	0.20	0.89	1.00	0.18
117501	CRTC	calcium ion binding	0.17	0.93	1.00	0.16

**Table 4.** SVM prediction performance using different biomarkers.

**Leave-one-out Estimates 20 markers**	**Microarray 14 markers**	**Microarray PSA only**	**Mass Spectrometry**
**Error**	14.71	25.49	16.67
**Recall**	80.77	75.00	85.51
**Precision**	89.36	75.00	83.10
